# Esophageal cancer in a young woman with bulimia nervosa: a case report

**DOI:** 10.1186/1752-1947-1-160

**Published:** 2007-11-29

**Authors:** Eric T Shinohara, Samuel Swisher-McClure, Michael Husson, Weijing Sun, James M Metz

**Affiliations:** 1 Department of Radiation Oncology, Hospital of the University of Pennsylvania, Abramson Cancer Center, Philadelphia, PA 19104, USA; 2 West Virginia University School of Medicine, Morgantown, WV 26506, USA; 3 Department of Pathology, Pennsylvania Hospital, Philadelphia, PA 19107, USA; 4 Department of Hematology Oncology, Hospital of the University of Pennsylvania, Abramson Cancer Center, Philadelphia, PA 19104, USA

## Abstract

Adenocarcinoma of the esophagus has increased dramatically within the United States and continues to have a poor prognosis despite aggressive treatment. Identifying potential risk factors is critical for the early detection and treatment of this disease. The present case report describes a very young woman who developed adenocarcinoma of the esophagus after only a brief history of bulimia. These findings suggest that even in very young patients, bulimia may represent a risk factor for adenocarcinoma of the esophagus.

## Introduction

In the past twenty-five years, the prevalence of esophageal adenocarcinoma has increased dramatically within the United States and it is now the most common histological type of esophageal cancer [1]. Despite advances in treatment, adenocarcinoma of the esophagus has a poor prognosis [2]. A recent study of patients with resectable disease demonstrated 5 year overall survivals of 81%, 51%, 14%, and 0% for stage I through IV respectively [3]. Previously implicated risk factors for esophageal adenocarcinoma include gastroesophageal reflux disease, tobacco use, obesity, and Barrett's esophagus. Prior reports have also suggested that chronic bulimia nervosa (BN) is a risk factor for the development of esophageal adenocarcinoma. Repeated microtrauma, due to vomiting, may contribute to the malignant transformation of the esophageal tissue. We report the case of a 27 year old female patient with a remote history of BN recently diagnosed with adenocarcinoma of the esophagus.

## Case presentation

A 27 year old female presented with a one year history of progressively worsening epigastric pain, reflux, and fatigue. She was initially treated with acid suppression therapy by her primary care physician, which temporarily relieved her symptoms. However, her symptoms became refractory to medication and she noted the onset of dysphagia. She reported a remote history of bulimia nervosa (BN) of approximately one year duration at the age of 17. She reported episodes of binge eating and self-induced vomiting, at least once a day. She denied any further history of bulimia since that time, which was corroborated by her mother. The patient reported smoking approximately 10 cigarettes per day since the age of 20, and had recently quit. She denied alcohol and drug abuse, and had no family history of malignancy.

On physical examination the patient's weight was 102 lbs (BMI 18.7). There were no physical findings suggestive of chronic bulimia such as dorsal finger calluses, dental erosion, or parotid enlargement. She denied recent weight loss. The patient had mild epigastric tenderness on palpation of the abdomen. The remainder of her examination was within normal limits. Routine laboratory tests were normal

**Figure 1:**
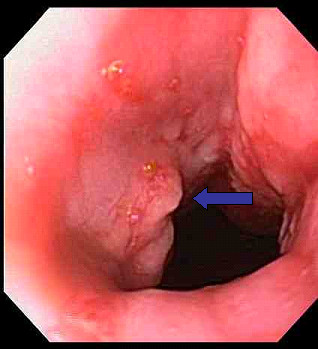
**Endoscopy**. Shown is a small 10 mm ulcerated lesion at the GE junction (blue arrow) seen on endoscopy.

**Figure 2:**
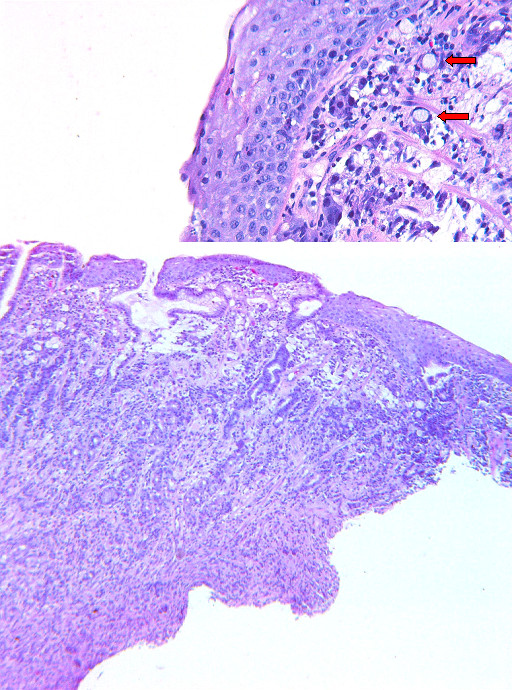
**Biopsy from endoscopy**. High power and low power images from endoscopic biopsy are shown above. Invasive adenocarcinoma and glandular metaplasia can be seen beneath the intact squamous epithelium on the lower power image. Signet rings (red arrows) are seen in the higher power image.

Upper gastrointestinal endoscopy revealed a 10 mm ulcerated lesion with diffuse erythema near the gastroesophageal (GE) junction (Figure 1). Biopsies from this area demonstrated poorly differentiated adenocarcinoma (Figure 2). Endoscopic ultrasound revealed a 20 mm × 20 mm, hypoechoic, non-circumferential mass at the GE junction with evidence of serosal invasion, but no nodal disease. CT scan revealed no evidence of nodal or metastatic disease. The patient was clinically staged as having T3 N0 M0 disease (Stage IIa).

The patient elected to undergo neoadjuvant chemoradiation with oxaliplatin, 5-FU, and Cetuximab with concurrent radiation to a dose of 5040 cGy. She had j-tube placement prior to treatment. She required one admission for fluids and nutritional support during treatment. Six weeks after completing neoadjuvant chemoradiation she underwent an Ivor Lewis esophagectomy, during which a right ovarian mass was noted and biopsied. Frozen section revealed metastasis and a right oophorectomy was performed. Pathology revealed signet ring cell adenocarcinoma of the GE junction and ovary, three positive gastric lymph nodes and three negative esophageal nodes. She then received adjuvant chemotherapy with epirubicin, cisplatin and capecitabine for six cycles. The patient was then followed regularly every three months with CT imaging. Interval evaluation at twelve months after diagnosis, five months after completing adjuvant chemotherapy, demonstrated no evidence of disease. Unfortunately, CT performed thirteen months after diagnosis demonstrated interval development of pleural effusions, ascities and a large pelvic mass, likely arising from the left ovary, consistent with recurrent metastatic disease.

## Discussion

The present case report demonstrates the importance of diagnosing esophageal cancer early, particularly in young patients, as advanced disease carries a devastating prognosis. Previous studies have demonstrated an associated between adenocarcinoma of the esophagus and reflux [4], the length and severity of reflux [5], and drugs which relax the lower esophageal sphincter [6]. These studies have suggested up to a 44 fold increase in the risk of developing adenocarcinoma of the esophagus with severe reflux and a 30–125 fold increase in risk in patients with Barrett's esophagus [7]. It appears family history is not strongly associated with the risk of developing esophageal cancer [8]. In the case of our patient there was no history of chronic reflux disease, with reflux symptoms only arising near the time of diagnosis. Furthermore, she had limited exposure to cigarettes and alcohol, which are more commonly associated with squamous cell carcinomas of the esophagus. Given the lack of other risk factors, it seems reasonable to consider her history of bulimia as a possible risk factor for her cancer. Similar to chronic reflux, bulimia may cause chronic irritation and trauma to the esophagus leading to dysplasia and ultimately tumorgenesis.

It is difficult to determine BN's exact prevalence due to changes in definition and difficulty in obtaining accurate responses in surveys, but it appears to be about 2% [9]. Esophagitis and Barrett's esophagus are known complications of BN [10], however only a few cases of esophageal cancer arising in patients with BN have been reported. Two cases of women in their 30's with a history of BN who developed esophageal cancer have been reported but no further details were provided [11]. Another case report described a young male patient with adenocarcinoma of the cervical esophagus who had a history of BN and alcohol abuse [12]. Endoscopy demonstrated extensive Barrett's esophagus and high grade dysplasia of the entire esophagus with superimposed candidial infection. A patient with longstanding BN who developed adenocarcinoma of the stomach has also been reported [13]. Another case report describes a 42 year old woman with a history of BN, without a history of smoking or drinking, who developed squamous cell carcinoma (SCC) of the distal esophagus [14]. These reports are summarized in (Table 1). The present case report describes a 27 year old female with a remote history of BN, which was of short duration, who was subsequently diagnosed with adenocarcinoma of the esophagus. This patient is unique for a number of reasons including her young age and the relatively short history of bulimia, which was significantly less severe than prior case reports.

**Table 1:** Summary of case reports describing patients with BN who developed esophageal cancer.

Author	Sex	Age	Location	Barrett's	Histology	Duration of BN (Years)
Walker, ES [13]	Female	61	Gastric (GE Junction?)	N/A	Adenocarcinoma	44
Navab, F [12]	Male	37	Cervical esophagus	Yes	Adenocarcinoma	Since High School
Buyse, S [14]	Female	42	Distal Esophageal	N/A	Squamous Cell Carcinoma	15
Present Report	Female	27	GE Junction	N/A	Adenocarcinoma	~1

## Conclusion

Studies suggest that there are long delays in the diagnosis of esophageal cancer [15]. Determining which factors put a patient at increased risk is critical as advanced disease has a poor prognosis. Esophageal cancer is most prevalent among older patients; however BN may represent an important risk factor in younger patients. Several prior case reports describe patients who were diagnosed at a young age (Table 1). This suggests that endoscopy may be warranted in younger patients with BN who present with new onset of persistent pain, weight loss, odynophagia or dysphagia. The course of the present patient suggests that even a remote, short history of BN may increase the risk for adenocarcinoma of the esophagus.

## Competing interests

The author(s) declare that they have no competing interests.

## Authors' contributions

ETS, WS and JMM were involved with the treatment on this patient.

ETS, SSM, JMM were involved in the data collection and drafting of the manuscript.

MH reviewed all pathological specimens.

All authors reviewed the final drafting of this manuscript.

## Consent

The authors would like to thank the patient for providing informed consent for the publication of this case report.
